# Call‐specific patterns of neural activation in auditory processing of Richardson’s ground squirrel alarm calls

**DOI:** 10.1002/brb3.1629

**Published:** 2020-04-19

**Authors:** Angela R. Freeman, James F. Hare, Heather K. Caldwell

**Affiliations:** ^1^ Laboratory of Neuroendocrinology and Behavior Department of Biological Sciences Kent State University Kent OH USA; ^2^ Department of Biological Sciences University of Manitoba Winnipeg MB Canada; ^3^ School of Biomedical Sciences Kent State University Kent OH USA

**Keywords:** amygdala, genes, immediate‐early genes, sciuridae, superior colliculi, vigilance

## Abstract

**Introduction:**

Richardson's ground squirrels use alarm calls to warn conspecifics about potential predatory threats. Chirp calls typically indicate high levels of threat from airborne predators, while whistle calls are associated with lower levels of threat from terrestrial predators. These types of calls primarily elicit escape behaviors and increased vigilance in receivers, respectively. While much is known about the neural mechanisms involved in the production of vocalizations, less is known about the mechanisms important for the perception of alarm calls by receivers, and whether changes in perceived risk are associated with unique patterns of neuronal activation. Thus, to determine whether alarm calls associated with different levels of predation risk result in differential neuronal activation, we used immunohistochemistry to identify and quantify c‐Fos immunopositive cells in brain regions important in stress, fear, danger, and reward, following alarm call reception.

**Methods:**

We exposed 29 female Richardson's ground squirrels (10 control, 10 whistle receivers, and 9 chirp receivers) to playbacks of whistles, chirps, or a no‐vocalization control. We then assessed neuronal activation via c‐Fos immunohistochemistry in 12 brain regions.

**Results:**

Ground squirrels receiving high‐threat “chirp” vocalizations had reduced neuronal activation in the medial amygdala and superior colliculus compared with controls. It is likely that changes in activity in these brain regions serve to alter the balance between approach and avoidance in turn promoting escape behaviors.

**Conclusions:**

Thus, we conclude that in Richardson's ground squirrels, these brain regions are important for the perception of risk resulting from receiving alarm calls and allow for appropriate behavioral responses by receivers.

## Graphical abstract

Richardson's ground squirrels use alarm calls to warn conspecifics about potential predatory threats. The “chirp” alarm call is thought to indicate high levels of threat, while the “whistle” is thought to indicate low levels of threat. Chirps typically elicit escape behaviors in receivers, which, we show, are supported by call‐specific neural activation patterns.

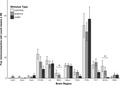

## 1 INTRODUCTION

Communication by group‐living animals facilitates a wide array of behaviors. The importance of vocal communication for mammals is supported by research suggesting that vocalizations can be costly as they can render the caller obvious, increase susceptibility to predation, or alert potential prey to the signaler's presence (Deecke, Ford, & Slater, 2005; Sherman, 1977). Nonetheless, vocal communication remains an efficient way to rapidly convey a wealth of information to receivers over distances.

Many of the neuronal mechanisms essential for vocal communication in mammals, particularly the production of these vocal signals, have been identified (Arriaga, Zhou, & Jarvis, 2012; Brudzynski & Bihari, 1990; Portfors, 2018; Shu et al., 2005). However, to understand the neural regulation of communication, mechanisms important for both production and perception of signals, and their relationship to behavior must be explored. The neural circuit involved in vocal signal perception involves the inferior colliculus for integrating auditory information, the medial geniculate to filter auditory information, and the auditory cortex, where learning and memory of these auditory signals likely occur (Asaba, Hattori, Mogi, & Kikusui, 2014; Heffner & Heffner, 1984; Portfors, 2018). The auditory cortex then projects to many brain regions, which can have specific impacts on behavior depending on the signals received (Banerjee & Liu, 2013). The perception of vocal signals can be lateralized in the auditory cortex, though in some cases this lateralization is context‐ and call‐specific (Geissler & Ehret, 2004; Heffner & Heffner, 1984; Sadananda, Wöhr, & Schwarting, 2008; Taglialatela, Russell, Schaeffer, & Hopkins, 2009). In regions outside the temporal cortex, lateralized responses during perception are largely absent (Geissler, Sabine Schmidt, & Ehret, 2013; Ouda, Jílek, & Syka, 2016).

In addition to activating regions in the auditory circuit, perceiving vocalizations can modulate neuronal activity in brain regions important for sexual behavior, parental behavior, stress, learning, affective salience, and reward (Ouda et al., 2016; Parsana, Li, & Brown, 2012; Pultorak et al., 2016; Sadananda et al., 2008; Willuhn et al., 2014). To date, much of our understanding of the brain areas involved in the perception of vocalizations has been explored in mice and rats. Both of these species can behaviorally discriminate between species‐specific call types, and this discrimination often correlates with differential activation or inhibition in specific brain regions (Neilans, Holfoth, Radziwon, Portfors, & Dent, 2014; Sadananda et al., 2008; Schwarting, Kisko, & Wöhr, 2018).

As would be predicted for most species, in mice and rats context strongly influences both the location and the degree of neuronal activation during auditory perception. For example, in female mice, both virgins and dams have differential activation in response to 50 and 22 kHz stimuli (Geissler et al., 2013), and the auditory cortex changes with exposure to, and recognition of ultrasonic calls (Fichtel & Ehret, 1999; Geissler, Schmidt, & Ehret, 2016; Liu, Linden, & Schreiner, 2006). Perception of calls associated with positive affect (i.e., 50 kHz ultrasonic vocalizations) results in increased activation of the frontal cortex (FCOR), nucleus accumbens (NAcc), and paraventricular nucleus of the thalamus (PVT; Pultorak et al., 2016; Sadananda et al., 2008), and decreased activation in the amygdala in rats (Parsana et al., 2012). In addition, perception of these calls is accompanied by increased dopamine release in the NAcc, which is thought to reinforce approach behavior by receivers (Willuhn et al., 2014). In contrast, the 22 kHz “aversive” or “alarm” call (Litvin, Blanchard, & Blanchard, 2007) results in increased activation in the perirhinal cortex, basolateral, central, and lateral amygdala, periaqueductal gray (PAG), hypothalamic and thalamic nuclei, and hippocampus of rats (Beckett, Duxon, Aspley, & Marsden, 1997; Neophytou et al., 2000; Ouda et al., 2016; Parsana et al., 2012; Sadananda et al., 2008). These regions are important for the regulation of fear and anxiety (Neophytou et al., 2000; Parsana et al., 2012), and thus, the varied behavioral responses observed after reception of 22 versus 50 kHz calls are modulated through differential activation of certain brain regions.

Richardson's ground squirrels (*Urocitellus richardsonii*) are social, colonial rodents that produce alarm calls alerting conspecifics to potential predatory threats. These squirrels have unique call types that are used in different contexts to convey information about those threats. Two of these calls, the “chirp” and “whistle,” are used by callers to indicate a high and low level of threat, respectively (Davis, 1984; Sloan, Wilson, & Hare, 2005). Generally, squirrels use “chirps” for airborne predators, and “whistles” for terrestrial predators, though there are a few exceptions (Davis, 1984; Hare, 1998a). In response, receivers exhibit a higher level of vigilance and are more likely to run to burrows in response to chirps than to whistles (Sloan et al., 2005). Richardson's ground squirrels are an excellent model for examining the neural mechanisms of perception of alarm calls due to the breadth of research which has examined behavioral responses to calls in varied contexts in the field (e.g., Hare, 1998b; Hare & Warkentin, 2012; Sloan et al., 2005; Swan & Hare, 2008a, 2008b; Wilson & Hare, 2003). In addition, while differences in rat and mouse strains are sometimes noted in communication work (Schwarting et al., 2018), free‐living Richardson's ground squirrels are outbred and behave similarly in widely dispersed populations (Davis, 1984; Downey, Jones, Quinlan, & Scrimgeour, 1994; Hare, 1998a). Since Richardson's ground squirrels exhibit robust behavioral responses to specific alarm call types, we posited that these call‐specific behavioral responses would be supported by call‐specific neuronal activation patterns. Thus, we used Richardson's ground squirrels to determine whether chirp and whistle alarm call types resulted in differential neuronal activation in brain regions of interest.

To assess neuronal activation, we used c‐Fos immunocytochemistry as an indicator of immediate‐early gene (IEG) activation. While a variety of IEGs exist (e.g., *Arc*, *Egr‐1*, *Npas4*), c‐Fos is commonly used due to a “high induction threshold” (Okuno, 2010). Furthermore, the c‐Fos cascade and its associated molecular tools are robust; c‐Fos immunocytochemistry has been reliably used as an indicator of neuronal activation in auditory perception studies in rats (Sadananda et al., 2008), mice (Fichtel & Ehret, 1999; Geissler et al., 2013, 2016), hamsters (Wallhäusser‐Franke et al., 2003), and marmosets (Miller, DiMauro, Pistorio, Hendry, & Wang, 2010). In these studies, c‐Fos immunoreactivity was not limited to the auditory cortex and can therefore reveal brain regions important in coordinated behavioral responses to auditory stimuli.

Given the ability of rodents to discriminate among vocalizations both by context and by call type, we hypothesized that alarm calls would elicit neuronal activation via c‐Fos IEG induction in brain regions that are important to the functional support of behavioral responses including the enhanced vigilance of receivers. We further based predictions on observations of neural activation in rats and mice following reception of different call types. Specifically, we predicted that chirp receivers would exhibit an increase in c‐Fos expression, compared with whistle receivers, in regions associated with stress (the paraventricular nucleus of the hypothalamus [PVN]), since stressful stimuli are known to elicit activation in the PVN in rodents (Martinez, Calvo‐Torrent, & Herbert, 2002); in regions associated with defense, such as the PAG (Deng, Xiao, & Wang, 2016); in regions associated with fear or aversion, such as the amygdala and hypothalamus, which show c‐Fos immunoreactivity in rats responding with locomotion (defense and avoidance) to aversive auditory stimuli (Beckett et al., 1997; Neophytou et al., 2000; Sadananda et al., 2008), and the superior colliculus (SCOL; Dean, Redgrave, & Westby, 1989) which shows c‐Fos immunoreactivity following aversive behavior in rats (Sandner et al., 1993); in regions that are important for anxiety (medial amygdala [MeA] and lateral septum [LS]), which show c‐Fos immunoreactivity correlated with anxiety‐like behavior in rats (Duncan, Knapp, & Breese, 1996). Furthermore, we predicted that chirp receivers might have increased c‐Fos expression in the regions important to reward and sensory processing, such as the PVT (Otis et al., 2019), since this region is important in the production of alarm vocalizations in rats (Ouda et al., 2016) and is also important for the perception of acoustic stimuli (Beckett et al., 1997). We further predicted that whistle receivers would show increased c‐Fos immunolabeling in the NAcc, since nonaversive vocalizations activated this region in rats (Sadananda et al., 2008), though both the NAcc and the thalamus have previously been implicated in fear learning and discrimination (Antoniadis & McDonald, 2006). We also examined the FCOR, since this region has call‐dependent activation in rats, with nonaversive calls eliciting more Fos‐like immunolabeling (Sadananda et al., 2008). However, the FCOR is also important for associative fear learning (Nakayama et al., 2015), and in gerbils, it shows increased c‐Fos expression in response to an auditory stimulus (Wallhäusser‐Franke et al., 2003). We therefore predicted that both call types, but not the no‐vocalization control, would activate neurons in the FCOR and the auditory cortex (AUD).

## 2 MATERIALS AND METHODS

Twenty‐nine female juvenile Richardson's ground squirrels were trapped at the Assiniboine Park Zoo in Winnipeg, MB (49°52′N, 97°14′W), using Tomahawk live traps (Tomahawk Live Trap Co.) baited with peanut butter (No Name™ Smooth Peanut Butter; Loblaws Inc). We used females in this study due to their abundance and lack of dispersal. All trials were completed between 9 July 2015 and 3 August 2015. All research with animals was conducted in accordance with the Canadian Council on Animal Care guidelines and approved by the University of Manitoba Fort Garry Campus Animal Care Committee (F13‐014/1), Assiniboine Park Zoo Research Ethics and Review Committee (2014‐A003), and Manitoba Conservation (Wildlife Scientific Permit WB14952).

### 2.1 Audio playback

Once trapped, Richardson's ground squirrels were transported in the live trap covered in a pillowcase to a building on site which housed acoustic isolation chambers (MAC‐2 Controlled Acoustical Environment Chambers; Industrial Acoustics Company Inc.). We then removed the pillowcase and placed the trap containing the squirrel into the chamber. A fan and light (15W incandescent, 122 lumens, 2700K) were on in the chamber for the entirety of the habituation and trial. After two hours of habituation, squirrels were presented with either a whistle call, a chirp call, or no stimulus as a control. The playback tracks were adjusted to play for 2 min and contained 49 evenly spaced chirp or whistle syllables (0.426 syllable/s call rate; Sloan & Hare, 2004; Warkentin, Keeley, & Hare, 2001). All calls were recorded in 2010 and came from a set of 112 total syllables from three individuals of unknown sex that were unfamiliar to the subjects. Exemplars were selected for their high signal‐to‐noise ratio and absence of other background calls. The playback unit (Honeytone Amplifier; Danelectro Co.) was situated within the acoustic isolation chamber, connected to an external SONY Minidisc Player (SONY MZ‐N707, SONY) and adjusted to play calls at ecologically appropriate sound pressure levels (80 ± 5 dB SPL_A_ at 1m).

### 2.2 Tissue collection

One hour after being presented with the stimulus, squirrels were removed from the chamber and humanely euthanized via intracardiac pentobarbital injection subsequent to isoflurane‐induced anesthesia. Subject brains were swiftly removed and post‐fixed in 4% paraformaldehyde before slicing at 50 μm on a vibratome in five serial sets. Tissue was then stored in cryoprotectant (50% v/v 0.1 M potassium phosphate buffer, 30% w/v sucrose, 1% w/v polyvinylpyrrolidone, 30% v/v ethylene glycol) at −20°C until immunocytochemistry for c‐Fos was performed.

### 2.3 c‐Fos immunocytochemistry

Tissue was washed six times in 1X phosphate‐buffered saline (PBS) for 10 min at room temperature to remove cryoprotectant. Then, tissue was processed as previously described (Dhakar, Rich, Reno, Lee, & Caldwell, 2012), using a 1:1,000 primary antibody dilution (sc‐52, rabbit polyclonal; Santa Cruz Biotechnology). After incubation in diaminobenzidine, sections were washed twice for 5 min in 1X PBS, mounted onto microscope slides, air‐dried, and then coverslipped.

### 2.4 c‐Fos enumeration

**FIGURE 1:**
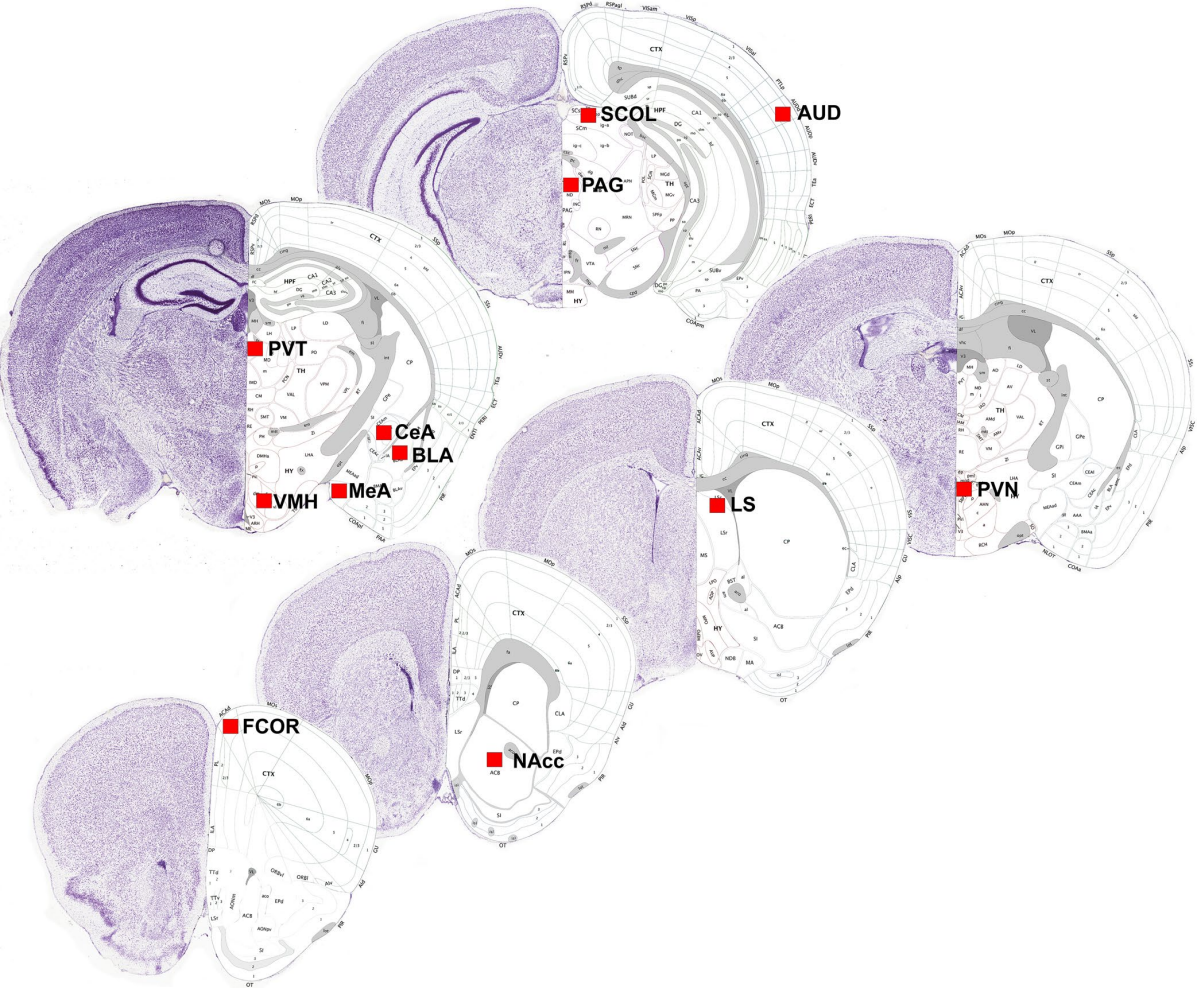
Twelve brain regions of interest. Locations where Fos‐immunoreactive cell counts were made are indicated with a red square. AUD, auditory cortex; BLA, basolateral amygdala; CeA, central amygdala; FCOR, frontal cortex; LS, lateral septum; MeA, medial amygdala; NAcc, nucleus accumbens; PAG, periaqueductal gray; PVN, paraventricular nucleus of the hypothalamus; PVT, paraventricular nucleus of the thalamus; SCOL, superior colliculus; VMH, ventromedial nucleus of the hypothalamus. Image credit: Allen Institute, http://atlas.brain‐map.org (Lein et al., 2007)

We selected 12 brain regions of interest (Figure 1), where we had a priori hypotheses concerning differences among groups, including the AUD, amygdala (central, medial, basolateral), LS, PVN, ventromedial hypothalamus (VMH), PAG, SCOL PVT, NAcc, and frontal association cortex (FCOR). We did not include the inferior colliculus due to difficulties obtaining caudal midbrain tissue from all animals (see Table 1 for number of animals providing data for each group and region). We enumerated all gray‐ to black‐stained cells (see Figure S1 for an example of included cells) within a 0.25 mm by 0.25 mm square for each region (Sadananda et al., 2008). All microscopy was completed using a Zeiss Axioplan 2 Microscope, and images were obtained using AxioVision 4.8.2. The location of the square was placed in the same area within each region, and placement of the square was selected by aligning the box with the scale bar placed on the image by the software program (Figure S1). This placement was chosen to reduce experimenter bias. We counted three sections for each animal and region and then used mean counts from each animal for analysis. We selected the “right” side of each section for counting Fos‐positive cells, though due to using free‐floating sections, we cannot establish which hemisphere(s) were enumerated. The experimenter was blind to the treatment group of each animal during enumeration. Regions were identified using anatomical landmarks (see Figure S2) using the Allen mouse brain atlas as a reference (Lein et al., 2007), and previously published Nissl‐stained sections of the Richardson's ground squirrel (Freeman, Hare, & Caldwell, 2019), since a Richardson's ground squirrel brain atlas does not exist. Therefore, some ground squirrel brain region boundaries may differ from those depicted in Figure 1. All images for enumeration were taken by ARF over several consecutive days to reduce variation.

**TABLE 1:** Number of animals providing data in each treatment group

	Control	Whistle	Chirp
AUD	8	10	7
Basolateral amygdala	10	9	8
Central amygdala	10	10	8
FCOR	10	10	9
LS	10	10	9
Medial amygdala	10	10	8
NAcc	10	10	9
PAG	9	8	6
PVN	10	10	9
PVT	10	10	9
SCOL	9	9	7
VMH	10	9	9

### 2.5 Data analysis

Data violated assumptions of normality; thus, nonparametric analyses were used. We examined differences between treatment groups for each brain region of interest using Kruskal–Wallis tests. We compared pairwise differences between groups using Dunn's tests with a false discovery rate (FDR) correction for multiple comparisons using the Benjamini–Hochberg procedure. We set *α* = 0.10 due to the inherent variability from working with a wild population from an uncontrolled environment. We report both FDR‐corrected and FDR‐uncorrected *p*‐values for Dunn's tests. All analyses were conducted using R 3.3.2 (R Development Core Team, 2016) and the Dunn.Test package (Dinno, 2017).

## 3 RESULTS

**FIGURE 2:**
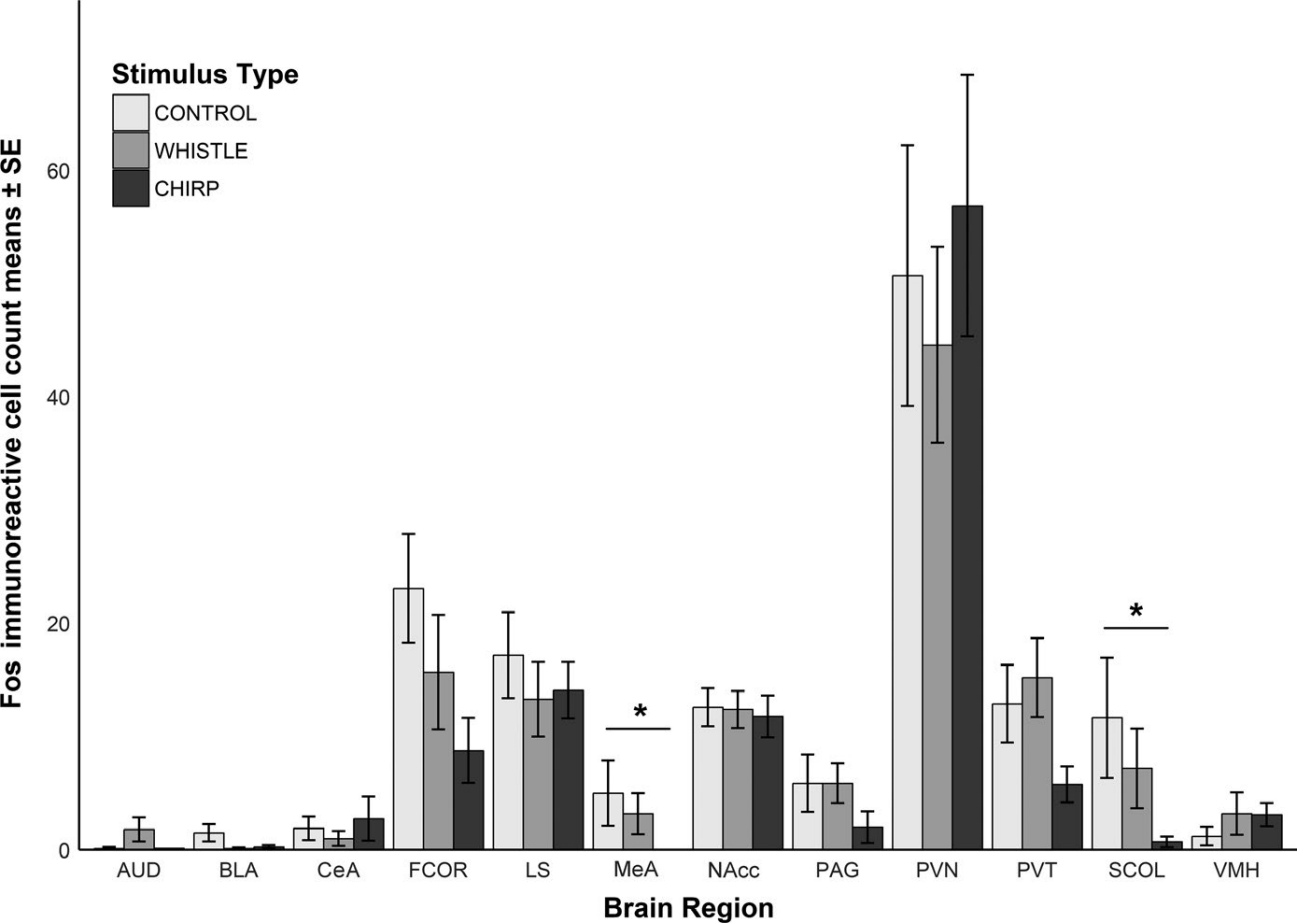
Distribution of Fos‐immunoreactive cells across brain regions by treatment (stimulus type). * indicates significant Dunn's test (*p* < .05). AUD, auditory cortex; BLA, basolateral amygdala; CeA, central amygdala; FCOR, frontal cortex; LS, lateral septum; MeA, medial amygdala; NAcc, nucleus accumbens; PAG, periaqueductal gray; PVN, paraventricular nucleus of the hypothalamus; PVT, paraventricular nucleus of the thalamus; SCOL, superior colliculus; VMH, ventromedial nucleus of the hypothalamus

**FIGURE 3:**
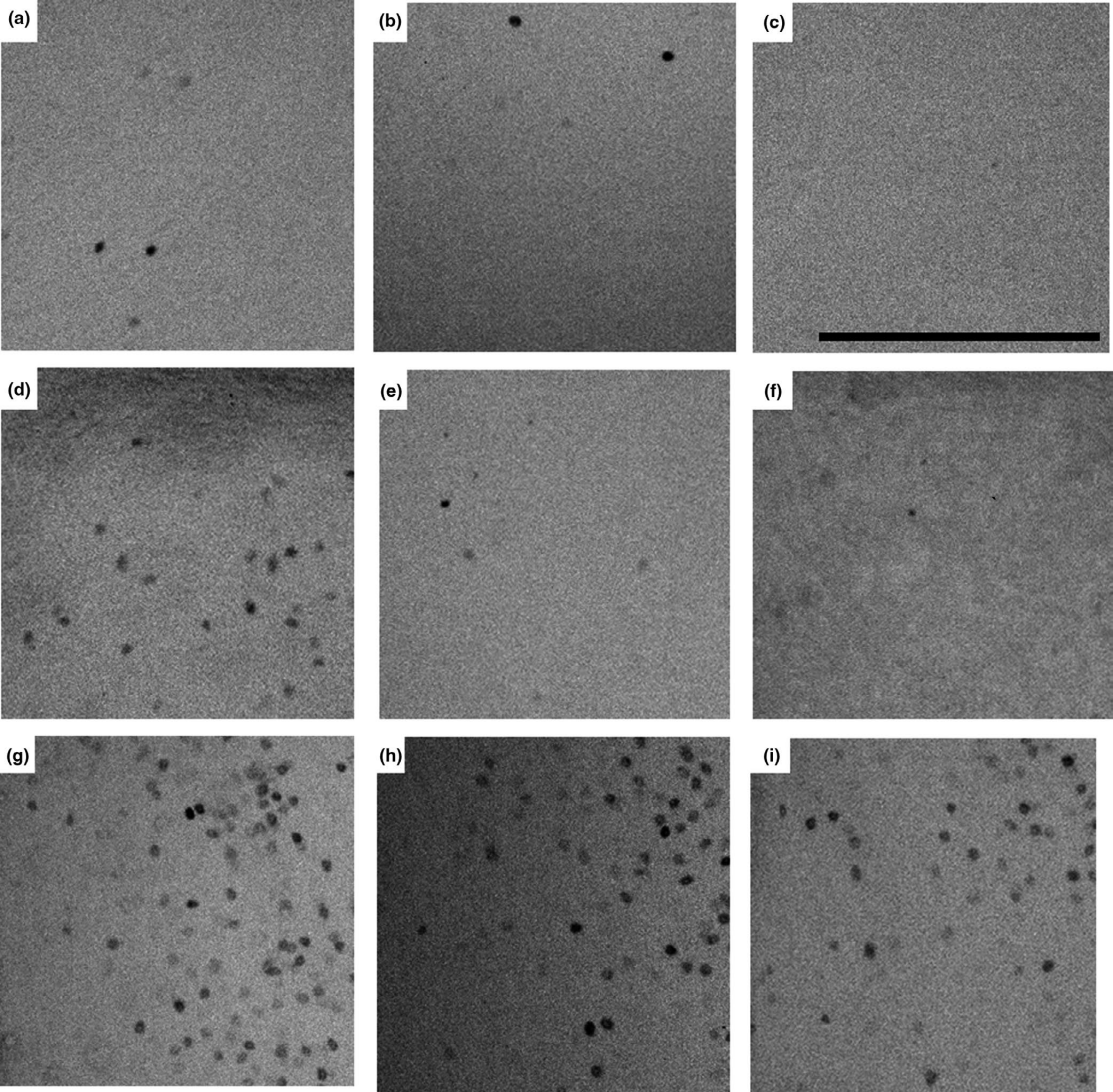
Representative micrograph selections where Fos‐immunoreactive cells were counted in the medial amygdala (a–c), superior colliculus (d–f), and paraventricular nucleus of the hypothalamus (PVN) (g–i). Chirp‐receiving animals (c, f) had significantly fewer Fos‐immunoreactive cells than control animals (a, d). Whistle‐receiving animals (b, e) had a moderate number of Fos‐immunoreactive cells, but did not significantly differ from control or chirp receivers after correction for multiple comparisons. All groups had many Fos‐immunoreactive cells in the PVN (controls, g; whistle receivers, h; and chirp receivers, i). Scale bar = 200 µm

Fos labeling was detected for each treatment or region of interest, though in some cases, we observed only a few stained cells within the selection box. All chirp receivers lacked Fos immunostaining in the medial amygdala (MeA). The Kruskal–Wallis tests identified significant differences in the MeA and SCOL (Table 2; MeA *χ* ^2^ = 5.15, *df* = 2, *p* = .08; SCOL *χ* ^2^ = 4.93, *df* = 2, *p* = .09) among the groups. A priori pairwise comparisons using Dunn's tests revealed that for these regions, individuals that were exposed to chirps had fewer immunoreactive cells compared with control animals (Figures 2 and 3; MeA: *Z* = −2.19, *p* = .042; SCOL: *Z* = −2.16, *p* = .047).

**TABLE 2:** Differences among groups in Fos‐immunoreactive cell counts as assessed by Kruskal–Wallis tests

Region	Control (mean ± *SD*)	whistle	Chirp	*χ* ^2^	*p*‐values
Auditory regions					
Auditory cortex	0.13 ± 0.35	1.8 ± 3.36	0.14 ± 0.38	1.52	.47
Fear/stress regions					
Central amygdala	1.90 ± 3.31	1.00 ± 2.11	2.75 ± 5.52	0.88	.64
Medial amygdala	5.00 ± 9.18	3.20 ± 5.69	**0** *	**5.15**	**.08**
Basolateral amygdala	1.50 ± 2.42	0.11 ± 0.33	0.25 ± 0.46	2.67	.26
Lateral septum	17.20 ± 12.04	13.30 ± 10.46	14.11 ± 7.64	0.56	.75
PVN	50.70 ± 36.41	44.60 ± 27.37	56.89 ± 34.69	0.64	.72
Ventromedial hypothalamus	1.20 ± 2.57	3.22 ± 5.59	3.11 ± 3.14	3.24	.20
Periaqueductal gray	5.89 ± 7.59	5.89 ± 5.23	2.00 ± 3.63	3.10	.21
Superior colliculus	11.67 ± 15.91	7.22 ± 10.54	**0.71 ± 1.25** *	**4.92**	**.09**
Danger/motivation/reward regions					
PVT	12.9 ± 10.88	15.2 ± 10.94	5.78 ± 4.74	4.24	.12
Nucleus accumbens	12.60 ± 5.32	12.40 ± 5.17	11.78 ± 5.56	0.27	.87
Information integration					
Frontal association cortex	23.10 ± 15.23	15.70 ± 16.01	8.78 ± 8.57	3.97	.14

* Significant compared with control, Dunn's test with FDR correction, *α* = 0.1.

Since comparisons were planned a priori in all measured brain regions, we also report the unadjusted *p*‐values in pairwise Dunn's tests as exploratory analyses (Table 3). These exploratory comparisons show marginal differences between the chirp and control groups in the VMH and FCOR, and between whistle and chirp groups for the MeA, SCOL, PAG, and PVT (Table 3).

**TABLE 3:** Pairwise comparisons of differences among controls, chirp receivers, and whistle receivers in 12 brain regions of interest

Region	Comparison	*Z*	*p*‐value	FDR‐adjusted *p*‐value
Auditory regions				
Auditory cortex	Control–chirp	0.07	.47	.47
	Control–whistle	−1.10	.14	.41
	Whistle–chirp	−0.98	.16	.25
Fear/stress regions				
Central amygdala	Control–chirp	−0.41	.34	.34
	Control–whistle	0.94	.17	.52
	Whistle–chirp	0.47	.32	.48
Medial amygdala	**Control**–**chirp**	**−2.20**	**.01** *	**.04** *
	Control–whistle	0.52	.30	.30
	**Whistle**–**chirp**	**−1.70**	**.04** *	.07
Basolateral amygdala	Control–chirp	−0.98	.16	.24
	Control–whistle	1.61	.05	.16
	Whistle–chirp	0.56	.29	.29
Lateral septum	Control–chirp	−0.48	.31	.47
	Control–whistle	0.74	.23	.69
	Whistle–chirp	0.23	.41	.41
PVN	Control–chirp	0.71	.24	.71
	Control–whistle	0.69	.49	.49
	Whistle–chirp	−0.03	.25	.37
Ventromedial hypothalamus	**Control**–**chirp**	**1.75**	**.04** *	.12
	Control–whistle	−1.21	.11	.17
	Whistle–chirp	0.53	.30	.30
Periaqueductal gray	Control–chirp	−1.42	.08	.12
	Control–whistle	−0.29	.39	.39
	**Whistle**–**chirp**	**−1.68**	**.05** *	.14
Superior colliculus	**Control**–**chirp**	**−2.16**	**.02** *	**.05** *
	Control–whistle	0.54	.30	.30
	**Whistle**–**chirp**	**−1.65**	**.05** *	.07
Danger/motivation/reward regions				
PVT	Control–chirp	−1.61	.05	.08
	Control–whistle	−0.34	.37	.37
	**Whistle**–**chirp**	**−1.94**	**.03** *	.07
Nucleus accumbens	Control–chirp	−0.50	.31	.92
	Control–whistle	0.14	.44	.44
	Whistle–chirp	−0.36	.36	.54
Information integration				
Frontal association cortex	**Control**–**chirp**	**−1.98**	**.02** *	.07
	Control–whistle	1.15	.19	.19
	Whistle–chirp	−0.87	.13	.19

* Significant *p* < .05.

Bold values are significant *p* < .05 prior to FDR‐correction.

## 4 DISCUSSION

Neuronal activation varied extensively among individuals; this may be due to the genetic and early life variability inherent in a wild population, or due to differences in behavioral responses during habituation and stimulus presentation. Contrary to our predictions, individuals that received chirps had significantly fewer immunoreactive cells in the MeA and SCOL compared with controls, which may be related to vigilance and avoidance behaviors. Subtle differences in neural activity among groups may be related to alarm call context and assessments of caller reliability. We conclude that reception of alarm calls, implying differential predation risk, results in call‐specific neural activation patterns.

### 4.1 Interpreting decreased Fos activity in the MeA and SCOL

We detected reduced activation in the MeA and the SCOL for chirp receivers. Both these regions are known for their roles in approach and avoidance (Arakawa, Arakawa, & Deak, 2010; Cohen & Castro‐Alamancos, 2010; Comoli et al., 2012), as well as emotion, fear, and social stress (Li, Maglinao, & Takahashi, 2004; Lupien, McEwen, Gunnar, & Heim, 2009; Matsuda et al., 1996), among other functions. The reduced neural activity (as measured by Fos immunoreactivity) in these regions compared with animals receiving no stimulus is unexpected, as studies examining immediate‐early genes usually detect increased immunoreactivity in animals receiving stimuli compared with baseline. A reduced amount of Fos immunoreactivity in chirp receivers likely indicates reduced neuronal activity in these regions.

It is unclear why Fos‐immunoreactivity levels are relatively higher in controls than the experimental groups’ levels in some regions. Individual variation in perception might be impacting the presence of Fos protein and overall gene expression. In some cases, changes in stimulus can result in reduced Fos expression (Burger et al., 2010), when increased expression is expected. On the other hand, reduced locomotion or diminished behavioral response to a stimulus can also be indicated by reduced Fos labeling, as in depressive‐like behaviors and adaptive coping in mice (Boucher et al., 2011). Finally, some forms of avoidance behavior might be mediated through neuronal inhibition, which can be detected by similar levels or slight reductions in Fos immunoreactivity compared with baseline conditions (Olazábal & Morrell, 2005). We did not assess behavior before capture, during habituation, or after the stimulus, so we cannot determine whether squirrels froze during the stimulus. However, squirrels were unable to perform typical avoidance behaviors (running, entering burrows; Hare & Warkentin, 2012) while receiving the stimulus.

In rats, activity in the MeA is essential for freezing responses after receiving predator cues (Müller & Fendt, 2006). For Richardson's ground squirrels, the reduction in activity in the MeA may reduce the likelihood of stationary behavior and promote escape or avoidance after receiving chirps. Alternatively, reduced activity might be due to the inability for ground squirrels to effectively escape while in an isolation chamber. In mice lacking oxytocin, plasma corticosterone concentrations were high after receiving a stressor, yet activation in the MeA was blunted compared with wild‐type mice (Mantella, Vollmer, Rinaman, Li, & Amico, 2004), suggesting that even in the absence of MeA activity, a robust stress response could occur. The SCOL also has a role in modulating responses to stressors, and in rats and mice, acute stressors can result in decreased activity in the SCOL (Matsuda et al., 1996; Sung et al., 2009), while corticosterone measures indicate a robust endocrinological response (Sung et al., 2009). Together, our findings suggest that reduced activity in these regions alters the balance of approach and avoidance in ground squirrels after receiving chirp alarm calls, which might promote the escape behaviors observed in natural populations. Future work should assess whether these patterns persist when ground squirrels exhibit call‐specific behavioral responses.

### 4.2 The stress response

We detected the largest number of Fos‐immunolabeled cells in the PVN (Figure 3). The PVN is a region important in the endocrine stress response, as corticotropin‐releasing hormone from the PVN modulates the release of adrenocorticotropic hormone from the anterior pituitary (Rivier & Vale, 1983). The high levels of Fos immunolabeling in the PVN among all groups, including the control, may be due to capture and trapping‐related stress (Delehanty & Boonstra, 2009); however, the large number of immunolabeled cells compared with other regions could also be impacted by neuron density. In previous work on this species investigating stress physiology, habituation to captivity (as measured by a reduction in fecal glucocorticoid metabolites) took several days (Hare et al., 2014). Elevated activity in the PVN in all groups might be a ceiling effect of ongoing capture‐related stress. We expected that chirps, as more “stressful” stimuli, would result in greater activation of cells in the PVN; however, we detected no significant differences among groups. Further investigation into the role of the PVN in predator vigilance and response behaviors is necessary, as all squirrels in the acoustic chamber had relatively high levels of PVN activation. In future work, a longer period of habituation to captivity may be necessary to resolve differences in activation in the PVN related to call‐specific stress.

### 4.3 Exploring assessments of reliability

While we had predicted that we would see differential activation in areas important in stress, anxiety, and fear, we detected no significant differences among groups in the PVN, central amygdala (CeA), basolateral amygdala (BLA), PAG, LS, and VMH after corrections for multiple comparisons. Similarly, after corrections we detected no significant differences among groups in the PVT, NAcc, or FCOR. As an exploratory approach, we report the uncorrected comparisons in our regions of interest, which suggest that subtle differences among groups may exist. Using this approach, differential expression between chirp receivers and controls was observed in the VMH and the FCOR, while differences between chirp receivers and whistle receivers were observed in the SCOL, MeA, PAG, and PVT. Notably, even with this exploratory approach, whistle receivers’ Fos labeling did not differ from controls, consistent with the lower response urgency conveyed by whistles relative to chirps (Warkentin et al., 2001).

One potential role of the subtle differences in these regions is that these regions’ activity is altered due to social assessment. Female ground squirrels were exposed to these calls in an acoustic isolation chamber, allowing us to determine how the perception of calls in isolation influenced neural activation. However, the context associated with these calls is known to affect receiver behavior, and presumably, neural activity. Richardson's ground squirrels are able to assess a caller's reliability, and individuals can learn to ignore squirrels that call repeatedly without the presence of a potential threat (Hare & Atkins, 2001). This lack of context, therefore, may have subtly altered neural activation in chirp and whistle receivers.

For example, activity during learning in the frontal association cortex is essential for encoding fear during conditioning in mice exposed to context and shock (Nakayama et al., 2015). For ground squirrels, context is also necessary for the expression of vigilance following reception of alarm calls, and repeated presentation of alarm calls from a single individual can lead to habituation in the absence of a natural predator (Hare, 1998a). The observed subtle differences in activity in the FCOR in chirp receivers compared with controls may provide a mechanism for Richardson's ground squirrels to learn to ignore unreliable callers (Hare & Atkins, 2001).

The PVT and PAG had fewer Fos‐labeled cells in the chirp group compared with the whistle group in our exploratory analysis. The PVT is thought to coordinate positive and negative emotions, sometimes discussed as the balance of “danger and reward” (Choi & McNally, 2017; Kirouac, 2015). Similarly, the PAG is a “coordinator of … defensive activities” (Fanselow, 1991). Altered activation in either of these regions can influence responses to predators (Blanchard, Williams, Lee, & Blanchard, 1981; Choi & McNally, 2017; Deng et al., 2016). Inactivation of PVT neurons in rats biased animals toward defense (freezing) or reward (food‐seeking) depending on context (Choi & McNally, 2017). For squirrels, the reduction in PVT activity may have a similar effect on behavior as observed in rats; with strongly “negative” calls enabling squirrels to shift from low‐vigilance behavior to high‐vigilance behavior, though further study is necessary to test this hypothesis. Like the reduction in the FCOR, a reduction in activity in the PVT and PAG may also be related to habituation or assessment of reliability of the caller, or due to contextual effects on neuronal activation. Examining neuronal activation while individuals are listening to multiple callers, or in the presence of a predator, may help determine whether habituation and learning are responsible for our observed reduction in Fos labeling.

## 5 CONCLUSIONS

The role of alarm calls in communicating the presence of threat and as a way for receivers to assess risk in group‐living animals is well understood. The neural mechanisms involved in reception of alarm calls are presumed to be similar among species under similar contexts (Oliveira & Faustino, 2017), though the neural substrates involved in the perception of “alarm calls” have largely been studied in laboratory rats (Litvin et al., 2007; Ouda et al., 2016; Parsana et al., 2012; Sadananda et al., 2008). Ground squirrels have a context‐specific alarm call repertoire and use different alarm call types to signal to receivers that can then assess their risk and respond accordingly (Hare, 1998a; Hare & Atkins, 2001; Sloan & Hare, 2008). Behavioral responses to these calls have been well‐studied, and this work demonstrates that calls conveying differential risk to receivers are associated with call‐specific patterns of neural activation. While we had predicted that Richardson's ground squirrels would have increased Fos immunoreactivity in brain regions important for predator defense, avoidance, stress, and others, we noted no increases and instead observed a reduced number of immunolabeled cells in the MeA and SCOL compared with controls. These regions are important in anxiety and processing information leading to aversive behavior, and differential activation in chirp receivers suggests that these regions are important in the perception of different alarm call types in Richardson's ground squirrels. Whether these patterns of neural activity vary in different contexts (e.g., in the natural environment) or not remains an open question. These neuronal changes may underlie the observed behavioral differences in receivers in response to alarm calls indicative of differential predation risk.

## CONFLICT OF INTEREST

The authors declare that there are no conflicts of interest regarding this work.

## AUTHOR CONTRIBUTIONS

All authors designed the study, wrote, and revised the manuscript. ARF conducted all experiments and data analysis. JFH and HKC provided funding and materials, and supervised the work.

## Supporting information

Fig S1

Fig S2

## Data Availability

The data that support the findings of this study are available from the corresponding author upon reasonable request.
