# Supplementary figures and images for: Call‐specific patterns of neural activation in auditory processing of Richardson’s ground squirrel alarm calls
Source: Brain Behav. 2020 Apr 19;10(6):e01629. doi: 10.1002/brb3.1629 (PMC7313678; doi:10.1002/brb3.1629)

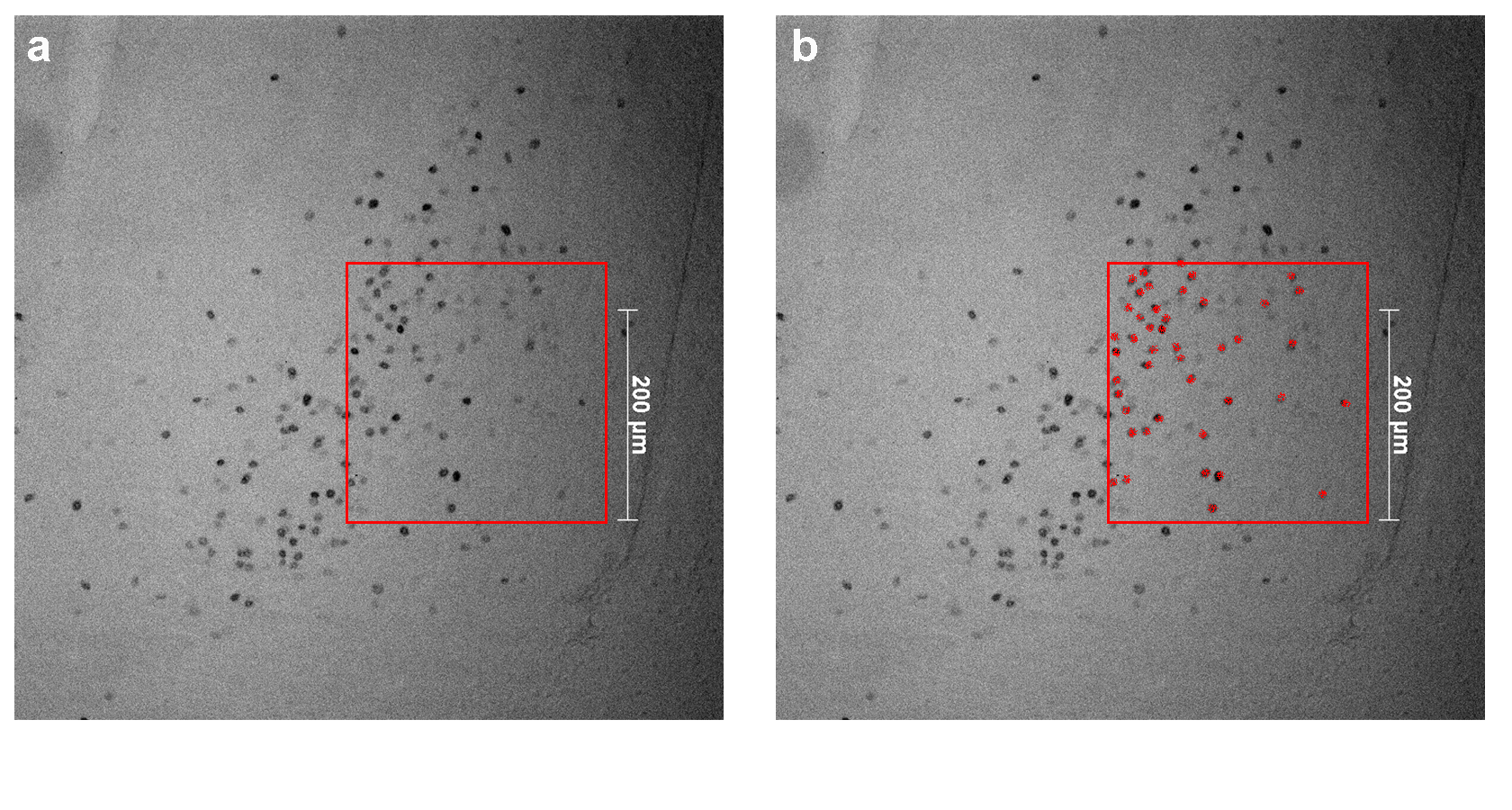

Supplement: Supplementary file 1 — Fig S1 [file BRB3-10-e01629-s001.tif]

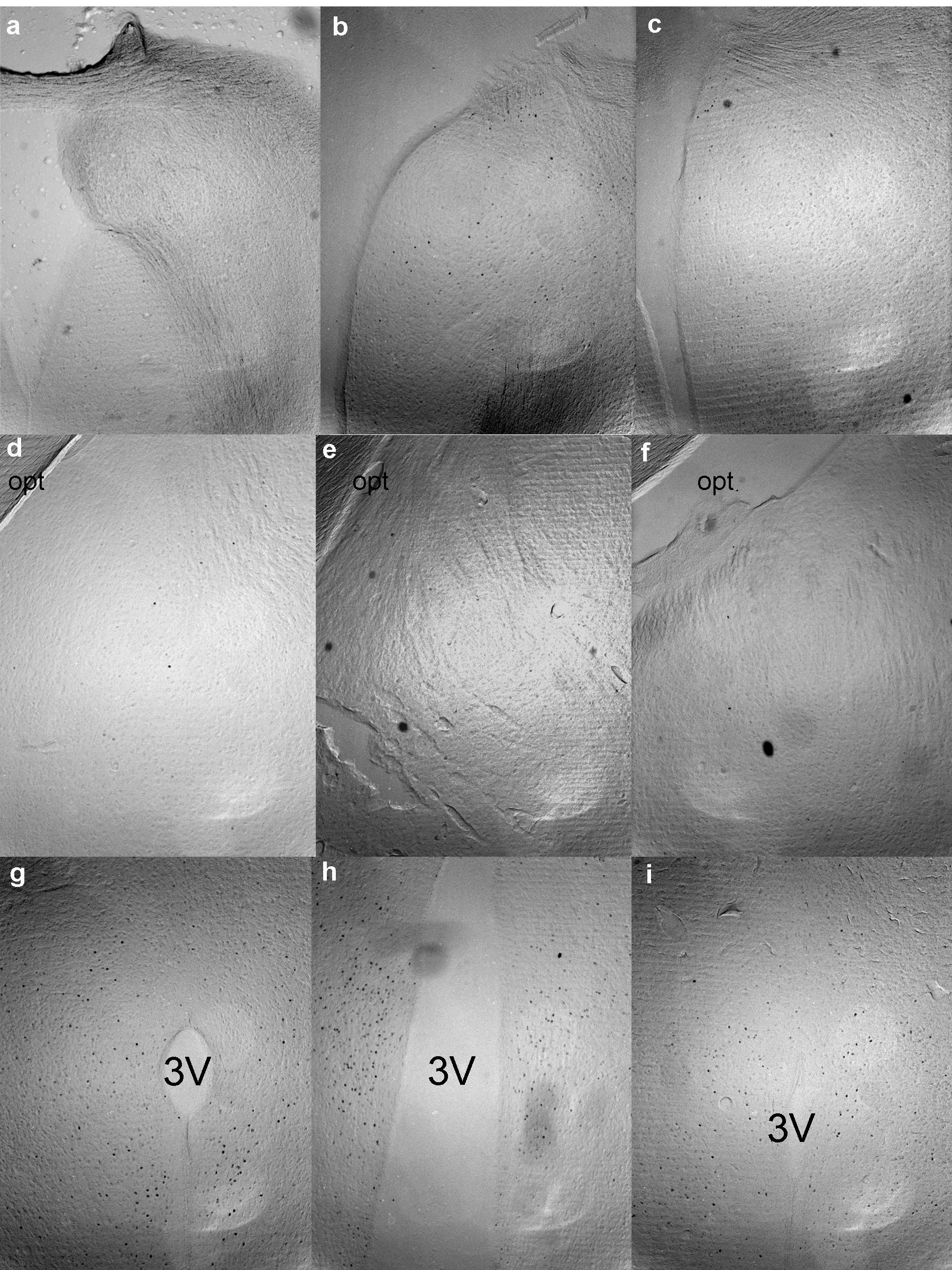

Supplement: Supplementary file 2 — Fig S2 [file BRB3-10-e01629-s002.tif]
